# Pegloticase immunogenicity: the relationship between efficacy and antibody development in patients treated for refractory chronic gout

**DOI:** 10.1186/ar4497

**Published:** 2014-03-04

**Authors:** Peter E Lipsky, Leonard H Calabrese, Arthur Kavanaugh, John S Sundy, David Wright, Marsha Wolfson, Michael A Becker

**Affiliations:** 1 1545 London Road, Charlottesville, VA 22901, USA; 2 Lerner College of Medicine, Cleveland Clinic Foundation, Cleveland, Ohio, USA; 3 University of California San Diego, La Jolla, CA, USA; 4 Duke University Medical Center, Durham, NC, USA; 5 Savient Pharmaceuticals Inc., Bridgewater, NJ, USA; 6 Formerly of Savient Pharmaceuticals Inc., East Brunswick, NJ, USA; 7 Rheumatology Section, University of Chicago, Chicago, IL, USA

## Abstract

**Introduction:**

The efficacy of pegloticase, a polyethylene glycol (PEG)-conjugated mammalian recombinant uricase, approved for chronic refractory gout, can be limited by the development of antibodies (Ab). Analyses from 2 replicate, 6-month, randomized controlled trials were performed to characterize Ab responses to pegloticase.

**Methods:**

Anti-pegloticase, anti-PEG, and anti-uricase Ab were determined by validated enzyme-linked immunosorbent assays. Ab titers were analyzed for possible relationships with serum pegloticase concentrations, serum uric acid (sUA) lowering, and risk of infusion reactions (IRs).

**Results:**

Sixty-nine (41%) of 169 patients receiving pegloticase developed high titer anti-pegloticase Ab (> 1:2430) and 40% (67/169) developed anti-PEG Ab; 1 patient receiving placebo developed high titer anti-pegloticase Ab. Only 14% (24/169) of patients developed anti-uricase Ab, usually at low titer. In responders, patients showing sustained UA lowering, mean anti-pegloticase titers at week 25 (1:837 ± 1687 with biweekly and 1:2025 ± 4506 with monthly dosing) were markedly lower than in nonresponders (1:34,528 ± 42,228 and 1:89,658 ± 297,797, respectively). Nonresponder status was associated with reduced serum pegloticase concentrations. Baseline anti-pegloticase Ab, evident in 15% (31/212) of patients, did not predict subsequent loss of urate-lowering response. Loss of sUA response preceded IRs in 44 of 56 (79%) pegloticase-treated patients.

**Conclusions:**

Loss of responsiveness to pegloticase is associated with the development of high titer anti-pegloticase Ab that increase clearance of pegloticase and are associated with a loss of the sUA lowering effect and increased IR risk. Pre-infusion sUA can be used as a surrogate for the presence of deleterious anti-pegloticase Ab.

**Trial registration:**

NCT00325195. Registered 10 May 2006, NCT01356498. Registered 27 October 2008.

## Introduction

Hyperuricemia creates the risk for deposition of urate crystals in tissues and increases the risk of developing the symptoms and signs of gout [1]. One new approach to urate-lowering is to convert urate to allantoin by administering the enzyme uricase, which is mutationally inactivated in humans. Although treatment with recombinant uricase is an attractive alternative, the enzyme has features that make it an ineffective pharmaceutical for chronic use, including poor solubility at physiologic pH, rapid clearance, and immunogenicity [2,3]. To overcome these obstacles, uricase can be coupled to polyethylene glycol (PEG), creating a pegylated molecule with reduced immunogenicity, enhanced solubility, and increased serum half-life [4,5]. Pegloticase is a mammalian recombinant uricase covalently conjugated to 10 (±1) strands of 10 kDa monomethoxy-PEG per uricase monomer [6]. Pegloticase has a serum terminal half-life of approximately 214 hours [7], and caused rapid persistent urate-lowering in response to repetitive administration for up to 6 months in approximately 40% of patients in two replicate, randomized, placebo-controlled trials (RCTs) [8,9]. Among patients in whom the initial urate-lowering response to pegloticase was lost subsequent to the first infusion, high titers of antibodies (Ab) against pegloticase were demonstrated. The objective of this report is to characterize the Ab response to pegloticase in patients with refractory chronic gout. The antigenic specificity of anti-pegloticase Ab was examined. In addition, the relationship between anti-pegloticase Ab titers and serum pegloticase concentrations, serum urate lowering capacity, and the risk of infusion reactions was also determined.

## Methods

### Study designs

Over the 6-month RCT [8] treatment period, patients received biweekly intravenous (IV) infusions consisting of either pegloticase 8 mg (biweekly cohort), pegloticase 8 mg alternating with placebo (monthly cohort), or placebo only. The primary endpoint was the number of patients with a treatment response defined as plasma urate (pUA) <6.0 mg/dL for ≥80% of the time during months 3 and 6 of the trial. Investigators were blinded to urate levels during the trials; consequently patients were maintained in the trials regardless of responder status, unless they encountered an adverse event that led to discontinuation from the study, were discontinued for other reasons, or withdrew consent. A total of 157/212 (74%) completed the RCTs; all patients withdrawing early were classified as nonresponders [8]. As previously reported [8], this study was carried out in accordance with the Helsinki Declaration, and received institutional review board approval at each site. Written informed consent and Health Insurance Portability and Accountability Act assurances were completed by each participant before enrollment.

### Antibody assays

Sera for measurement of Ab were collected at baseline and before infusions at weeks 3, 5, 9, 13, 17, 21, and 25 [9]. Ab directed against pegloticase, PEG, and uricase were measured using validated ELISA (see Additional file 1).

### Serum pegloticase levels

Blood samples were collected at baseline, before each infusion, at 1 and 7 days after the week-9 and week-21 infusions, at 7 days after the week-11 and week-23 visits, and at the final study visit for measurement of serum pegloticase concentrations. Samples for determination of trough pegloticase concentrations were drawn immediately before the pegloticase infusion and those for peak pegloticase concentrations were drawn approximately 2 hours following infusion completion. An enzymatic/fluorescence assay was used to quantitate pegloticase concentrations in serum (see Additional file 1). The lower limit of detection of serum pegloticase was 0.6 μg/mL.

### Statistics

All statistical calculations, including deriving means and SD, categorical data tests (that is, Chi square or Fisher’s exact test), and correlation analysis based on Pearson statistics were conducted with SAS 9.3 (Cary, NC, USA).

## Results

### Responder status and changes in serum uric acid (sUA)

**Figure 1:**
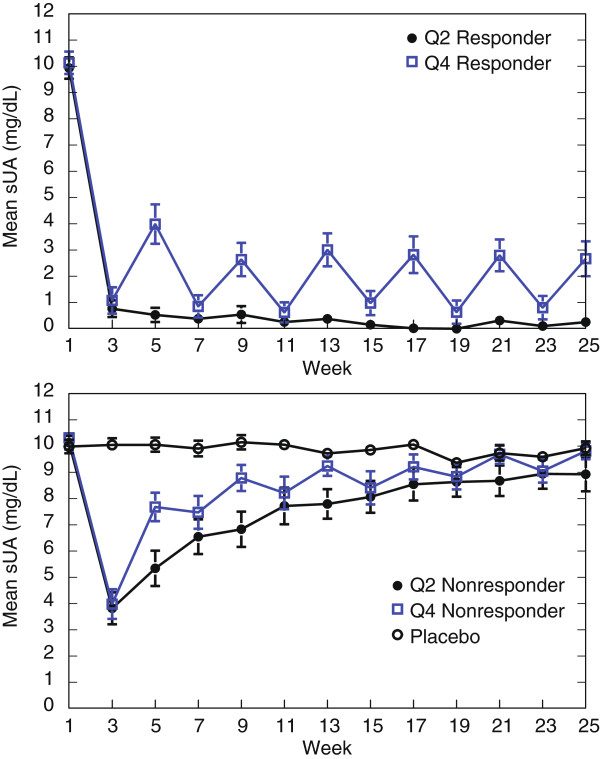
Mean serum uric acid (sUA) levels in responders (top panel) and nonresponders (bottom panel). Values for placebo-treated patients are shown in the graph of nonresponders.

In the modified intent-to-treat (mITT) population, 36 of 85 patients (42.4%) in the biweekly pegloticase cohort and 29 of 84 patients (34.5%) in the monthly pegloticase cohort were classified as responders [8]. The remaining 147 patients, including all 43 patients in the placebo cohort, and all 55 patients who did not complete the study, were classified as nonresponders. These protocol definitions of responder and nonresponder were retained in the current analyses. In all pegloticase-treated patients, sUA declined rapidly after the first infusion, and mean sUA remained at <2 mg/dL throughout the treatment period among responders to biweekly pegloticase (Figure 1). Responders to monthly pegloticase showed sUA reductions after each infusion of active treatment, but a *saw-tooth* pattern was apparent with increased sUA after the alternating placebo infusion. Although nonresponders to pegloticase also achieved a prompt sUA reduction after the first infusion, a loss in urate-lowering efficacy was seen relatively early after treatment initiation (Figure 1). Mean time to loss of uric acid (UA) response among nonresponders in both dosing cohorts was approximately 6 weeks.

### Anti-pegloticase Ab titers

Among RCT participants treated with pegloticase, 89% had measurable anti-pegloticase Ab titers during ≥1 study visit. An empirical approach was employed in an attempt to determine clinically relevant anti-pegloticase Ab titers. This involved a determination of the titer that most effectively discriminated between responders and nonresponders. This approach suggested that a titer >1:2430 might be clinically relevant. Using a titer >1:2430, 69/169 pegloticase-treated patients (41%) developed high anti-pegloticase Ab levels (38/85 [45%] in the biweekly group and 31/84 [37%] in the monthly group).

**Figure 2:**
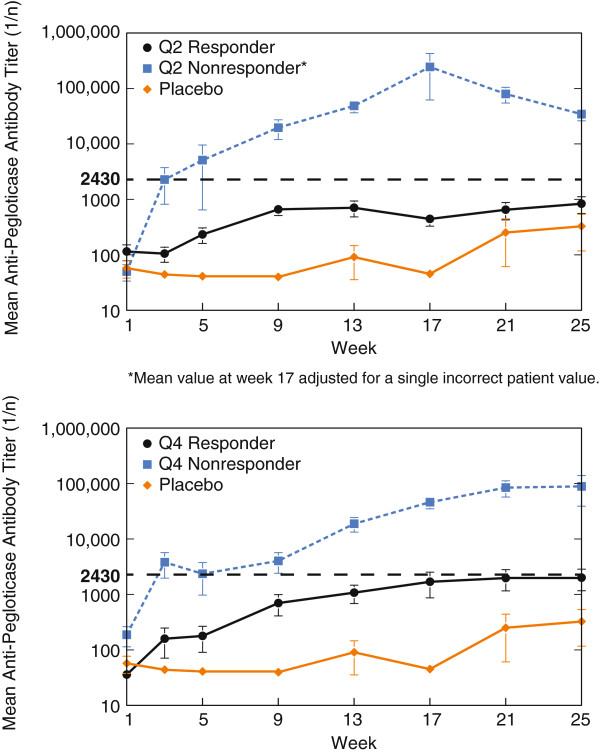
Mean anti-pegloticase Ab titers over time among serum uric acid (sUA) responders and nonresponders (and placebo-treated patients) receiving biweekly (top panel) and monthly (bottom panel) pegloticase.

For UA responders, mean anti-pegloticase Ab titers were <1:2430 at all study visits (Figure 2), whereas mean anti-pegloticase Ab titers in nonresponders rose to >1:2430 by the week-4 visit. Importantly, only 2/36 responders in the biweekly pegloticase cohort and 5/29 responders in the monthly pegloticase cohort manifested an anti-pegloticase Ab titer >1:2430 at any time during the trials, compared with 34/36 and 24/29 responders, respectively, who had an anti-pegloticase Ab titer ≤1:2430 at all time points (Additional file 2: Figure S1).

These data strongly suggest that an anti-pegloticase Ab titer >1:2430 was generally able to distinguish responders from nonresponders. Notably, when all samples were analyzed, correlation (*R*^2^ = 0.073, *P* <0.0001) between anti-pegloticase Ab titers and sUA was found. (Additional file 3: Figure S2).

Among placebo-treated patients, 4/43 had anti-pegloticase Ab at baseline and 8/43 developed anti-pegloticase Ab during the trials. In general, Ab titers were very low in patients treated with placebo and no pattern of increasing titers over time was apparent.

### Ig heavy chain isotype of anti-pegloticase Ab

**Figure 3:**
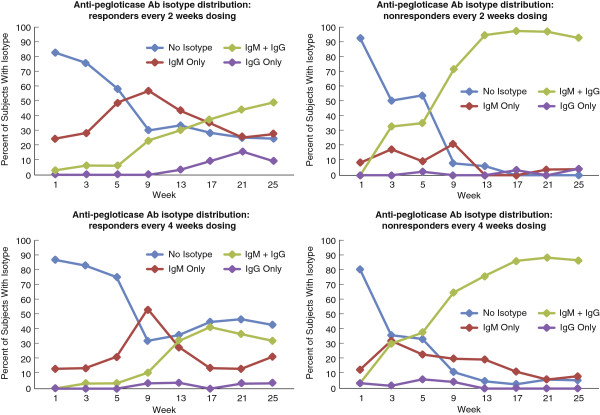
Percentage of patients with immunoglobulin (Ig)M and/or IgG anti-pegloticase antibodies (Ab) over time for responders and nonresponders receiving biweekly and monthly pegloticase.

For patients receiving biweekly pegloticase, responders produced mostly IgM Ab during the first 2 months of treatment, with the number of patients developing immunoglobulin (Ig)G (either with IgM or alone) increasing from week 9 to the end of the study (Figure 3). In comparison, nonresponders to biweekly pegloticase already produced both IgM and IgG by week 3, and most nonresponders produced both IgM and IgG Ab by the second half of the study. Responders and nonresponders receiving monthly pegloticase showed isotype patterns similar to those for their counterparts treated biweekly. Among patients who received placebo and developed anti-pegloticase Ab, 9/11 produced IgM only.

### Patients with positive anti-pegloticase Ab titers at baseline

Fifteen percent (31/212) of patients (including 4 from the placebo group) had positive anti-pegloticase Ab titers at baseline. Within the subgroup of 27 patients with baseline anti-pegloticase Ab treated with pegloticase during the RCTs, 12 (44%) met the criteria for a UA responder and 15 (56%) were nonresponders. These proportions are consistent with the UA response rates in the full study population described above. Mean baseline anti-pegloticase Ab titers were generally low (1:100 ± 193 in eventual responders and 1:198 ± 546 in eventual nonresponders) and were predominantly IgM. Among baseline Ab positive subjects, subsequent Ab titers were similar to that manifested by the full study cohort. Thus, for example, at the week-25 assessment, anti-pegloticase Ab titers were approximately 10-fold higher in nonresponders, albeit with wide variability (1:377 ± 1,143 in responders and 1:4022 ± 12,517 nonresponders).

### Specificity of anti-pegloticase Ab

Pegloticase, the immunogen in this study, is a recombinant mammalian uricase covalently coupled to 10 ± 1 strands of 10 kDa monomethoxy-PEG per uricase monomer [6]. Therefore, anti-pegloticase Ab could have been directed to the uricase protein or the PEG moiety or both. To determine the specificity of the anti-pegloticase Ab, a variety of PEGylated proteins, including pegloticase, were tested in a drug competition assay (Table 1). Soluble pegloticase reduced binding by 70%. PEG-asparaginase and PEG-catalase competed with pegloticase to a similar extent, whereas PEG-chymotrypsin and PEG-subtilisin were modestly less effective. PEG-superoxide dismutase competed at a reduced level although competition was still apparent. No competition was noted with the control proteins.

**Table 1:** Competition by pegylated and control proteins for binding of anti-pegloticase Ab

**Protein (200 μg/mL)**	**Low positive control serum**		**High positive control serum**	
	**Mean, A**_**450nm**_	**Difference, %**	**Mean, A**_**450nm**_	**Difference, %**
No protein	0.349		0.959	
Pegloticase	0.089	−74.5	0.254	−73.5
PEG-asparaginase	0.102	−70.8	0.326	−66.0
PEG-catalase	0.129	−63.0	0.289	−69.9
PEG-chymotrypsin	0.159	−54.4	0.491	−48.8
PEG-subtilisin	0.158	−54.7	0.423	−55.9
PEG-superoxide dismutase	0.244	−30.1	0.646	−32.6
Lysozyme/propylene oxide	0.324	−7.2	0.913	−4.8

PEG, polyethylene glycol.

To examine the specificity of the anti-pegloticase Ab response in further detail, anti-PEG Ab were measured. A total of 69/212 patients developed anti-PEG Ab and 64 (93%) of these were UA nonresponders (33 nonresponders receiving biweekly infusions and 31 nonresponders receiving monthly infusions). Additionally, three patients who qualified as UA responders and two placebo-treated patients developed anti-PEG Ab. Mean anti-PEG titers (Additional file 4: Figure S3) paralleled the pattern and time course of the mean anti-pegloticase titers.

Ab against the uricase portion of pegloticase (Additional file 5: Figure S4) were detected in 24 of the 212 patients (11%) in the mITT population. No placebo-treated patient developed anti-uricase Ab. With a single exception, no patient developed anti-uricase Ab before week 13. Of the 24 patients who developed anti-uricase Ab, 13 had only a single positive result, and 5 had Ab detected only at the final visit. Of the total of 40 samples that were positive for anti-uricase Ab, only 7 were found in samples with a simultaneous anti-pegloticase Ab titer of ≤1:2430. Ab that inhibited uricase activity were extremely rare, with only one possibly positive result noted in a patient who did not have Ab detected in the uricase ELISA assay.

### Anti-pegloticase Ab titers and pegloticase levels

**Figure 4:**
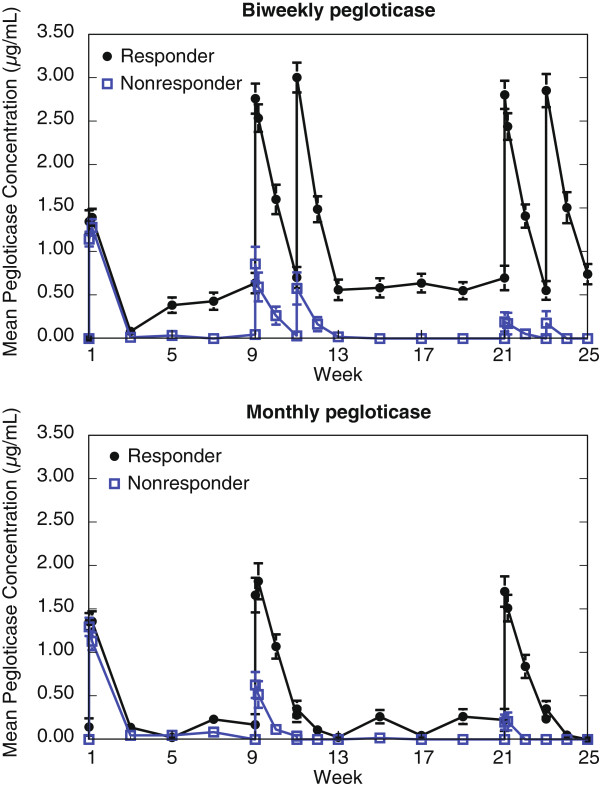
Mean serum pegloticase concentrations in responders and nonresponders receiving pegloticase biweekly (top panel) or monthly (bottom panel).

The mean peak serum pegloticase concentration was 1.4 ± 0.1 μg/mL measured 2 hours after the first dose in responders (n = 36) treated with the biweekly regimen (Figure 4). This cohort appeared to reach steady state by the next sampling time at week 9; all subsequent peak concentration values were in the range of 2.8 to 3.0 μg/mL. Nonresponders to biweekly infusions (n = 49) had peak pegloticase levels similar to the responder cohort after their first dose (1.1 ± 0.1 μg/mL), but concentrations declined steadily to a mean of 0.2 ± 0.1 μg/mL following the final dose and only 2/25 patients had detectable values after the final dose. Mean trough values ranged from 0.6 to 0.7 μg/mL for biweekly responders and below 0.05 μg/mL for biweekly nonresponders at all time points.

This pattern of pegloticase pharmacokinetics was similar for patients receiving monthly infusions. Mean peak pegloticase levels were similar for responders and nonresponders after the first dose (1.4 ± 0.1 μg/mL versus 1.3 ± 0.1 μg/mL, respectively). Final visit peak concentrations were 1.7 ± 0.2 μg/mL for monthly responders and 0.2 ± 0.1 μg/mL for monthly nonresponders. Final visit mean trough values were 0.2 ± 0.1 μg/mL for monthly responders and undetectable for the monthly nonresponders.

### Relationship between the time of UA rise and Ab response

**Figure 5:**
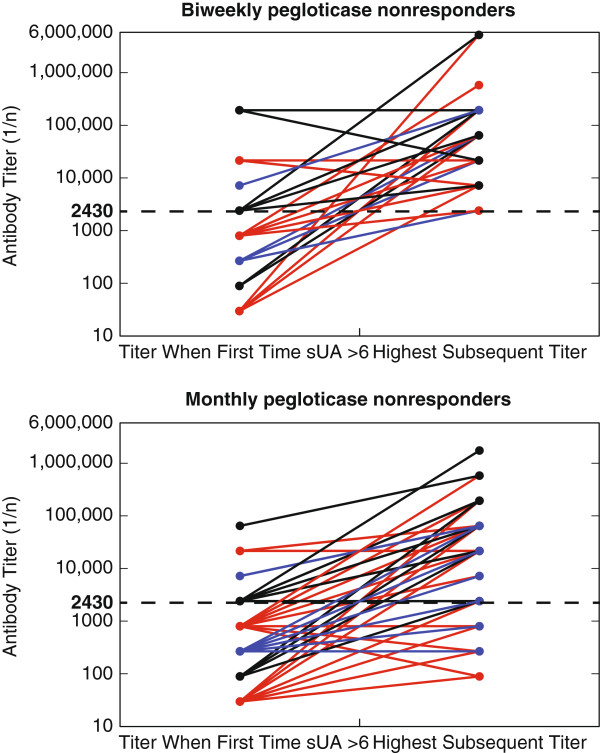
**Anti-pegloticase antibody (Ab) titer when serum uric acid (sUA) first exceeded 6 mg/dL and the highest titer subsequently detected during the randomized controlled trial.** Both sUA and Ab titers were measured in the same samples from patients defined as nonresponders.

As previously noted (Additional file 3: Figure S2), there was a significant correlation (*P* <0.0001) between sUA and the anti-pegloticase Ab titer, but the *R*^2^ value was only 0.073, suggesting that additional factors affected sUA levels. Moreover, it was noted that the titer of anti-pegloticase Ab at the time the sUA first rose to >6 mg/dL was variable, with many patients exhibiting low Ab titers at the time that the urate-lowering efficacy was lost, regardless of whether pegloticase was administered biweekly or monthly (Figure 5). However, most patients who lost their sUA-lowering response eventually developed high titers of anti-pegloticase Ab (Figure 5). The pattern of anti-pegloticase Ab titers and loss of sUA over time is shown in individual patients in Additional file 6: Figure S5.

### Ab titers, UA, and risk of infusion reactions (IRs)

IRs were reported in 22 patients receiving biweekly pegloticase, 34 patients receiving monthly pegloticase, and 2 patients receiving placebo [9]. IRs were more common among patients who developed high-titer (>1:2430) anti-pegloticase Ab; 38/56 or 68% of the pegloticase-treated patients with IRs also had high titer Ab. Similarly, the majority of patients with IRs had elevated sUA concentrations consistent with loss of efficacy of pegloticase. For example, among patients treated with the biweekly dose who experienced IRs (n = 22), 20 patients had sUA >6 mg/dL before the first IR (one patient had an IR with the first dose and one patient had an IR with an sUA concentration <6 mg/dL). Among patients treated with monthly pegloticase (n = 34), 24 had sUA >6 mg/dL before the first IR.

## Discussion

PEGylation of a recombinant mammalian uricase was successfully employed to develop a soluble drug, pegloticase, with a prolonged serum half-life and robust urate-lowering efficacy. As uricase is not expressed in humans, it can be considered a foreign protein, and as such, would be expected to elicit Ab formation routinely, as is observed after the administration of a non-pegylated uricase, rasburicase [10]. However, the immunogenicity of pegloticase appeared to be altered by pegylation, in that even though 89% of subjects generated measurable anti-pegloticase Ab, only 41% developed high titer Ab with the capacity to affect drug levels.

PEGylation is known to alter the immunogenicity of proteins [5]. However, the PEG moiety of the complex can itself become immunogenic resulting in the formation of Ab reactive against the PEG portion of the molecule [11]. Multiple lines of evidence support the conclusion that the PEG moiety is the primary target of anti-pegloticase Ab. First, competition assays demonstrated that Ab binding to pegloticase was significantly reduced by multiple PEGylated proteins. These results are consistent with other reports on the specificity of anti-pegloticase Ab [12,13]. Differences in the degree of competition among the substituted PEGylated proteins appeared to depend on the molecular weight of the competitor molecule as well as the extent of PEGylation. Larger proteins with more PEG strands competed more effectively than smaller proteins with fewer PEG strands, suggesting a multiplicity of soluble PEG molecules is required to compete effectively with Ab binding to pegloticase bound to the ELISA plate.

Anti-PEG Ab developed contemporaneously with anti-pegloticase Ab and the titers, albeit lower, paralleled the anti-pegloticase titers. The lower anti-PEG titers appeared to relate to differences in the assay such that interference by pegloticase in the test sera markedly reduced Ab titers. Despite this, a similar pattern was apparent for anti-pegloticase and anti-PEG Ab, thus supporting the results of the competition assays and previously published findings [12]. Moreover, the occurrence and titers of anti-PEG Ab were associated with a decrease in serum pegloticase content (unpublished data). These results are all consistent with the conclusion that most anti-pegloticase Ab are directed to the PEG portion of the molecule.

In contrast to Ab to the PEG portion of pegloticase, Ab to uricase were infrequent and, when present, usually developed after considerable exposure to pegloticase and in subjects with high titers of anti-pegloticase Ab. Previous data suggest that recombinant uricase does not competitively inhibit binding of anti-pegloticase Ab to pegloticase [13], indicating that anti-uricase and anti-pegloticase Ab represent separate specificities of Ab binding. Although anti-uricase Ab were more frequent in nonresponders, the timing of their occurrence, usually after the UA response was lost, suggests that they were unlikely to be the cause of the loss of responsiveness, but rather secondary to prolonged exposure to pegloticase in individuals who already had developed high titers of anti-pegloticase Ab. This conclusion is supported by the finding that measurable anti-uricase Ab were absent in many nonresponders. Notably, no evidence was found to support the possibility that anti-uricase Ab affected the function of pegloticase.

Two lines of evidence suggest that anti-pegloticase Ab affected pegloticase pharmacokinetics resulting in increased clearance and reduced drug concentrations to sub-therapeutic levels. First, the development of high-titer anti-pegloticase Ab in nonresponders was associated with reductions in peak and trough serum pegloticase concentrations. Second, the anti-pegloticase Ab had no direct inhibitory effect on pegloticase activity. Together, these data indicate that the major effect of clinically relevant anti-pegloticase Ab is to alter the pharmacokinetics of pegloticase.

Anti-PEG Ab have been reported with administration of other PEGylated agents. For example, anti-PEG Ab were closely associated with the rapid clearance of PEG-asparaginase in a subgroup of patients treated for acute lymphoblastic leukemia [11]. In contrast, no correlation was evident between anti-PEG Ab and serum asparaginase activity in control patients who received unmodified asparaginase. Similarly, anti-PEG Ab have been associated with enhanced blood clearance of PEG-modified liposomes and nanoparticles [14-16]. These findings are all consistent with the current results indicating that anti-PEG Ab altered the pharmacokinetics of pegloticase.

The relationship between the time of loss of the sUA lowering effect and the contemporaneous development of high titer Ab was not immediately apparent while the clinical trials were ongoing. Indeed, many nonresponders had low-titer Ab at the time urate-lowering efficacy was lost, although these patients subsequently developed higher titer Ab. At the time of the loss of the sUA response, it is likely that pegloticase was actually binding anti-pegloticase Ab, making it impossible to detect the Ab in the serum samples. Subsequently, as the Ab titers rose, the capacity of the antigen to bind Ab was superseded, and anti-pegloticase Ab could be detected in the serum. Importantly, the results imply that measuring Ab levels is not useful for making treatment decisions about continuing pegloticase treatment.

Nonresponders produced both IgG and IgM Ab, whereas responders tended to produce more IgM Ab and for a longer period of time. This suggests that nonresponders have a greater likelihood of developing T-cell dependent responses with associated increases in Ab titers and Ig heavy-chain isotype switching [17]. Despite this, it was not possible to determine whether IgG or IgM Ab mediated the loss of responsiveness. Although there was a tendency for the development of IgG Ab to parallel the loss of responsiveness, a number of subjects clearly lost responsiveness in the absence of IgG Ab.

Fifteen percent of patients had anti-pegloticase Ab at baseline, presumably against the PEG portion of the molecule. In most of these patients the Ab titer was low and was predominantly IgM. Naturally occurring anti-PEG Ab, predominantly IgM isotype, were initially reported by passive hemagglutination in 0.2% of healthy individuals and 3.3% of allergic patients nearly three decades ago [18]. More recent studies have found anti-PEG Ab in 4% to 25% of healthy blood donors, and have identified both IgG and IgM isotypes [11,19]. The reason for anti-PEG Ab in the general population remains to be determined, but may relate to enhanced assay sensitivity with ELISA methodology, or more likely, to increased exposure to PEG-containing products in cosmetics, processed foods, and pharmaceuticals [2,11,20,21]. In the present study, there was no evidence that the presence of anti-pegloticase Ab at baseline was related to risk of nonresponse to pegloticase.

To determine whether specific patient characteristics were associated with an increased likelihood of developing anti-pegloticase Ab, a number of demographic characteristics including age, gender, body mass index, and renal function were examined. The only one of these that was significantly associated with responsiveness was age. For example, 50% of subjects who were 60 years of age or older were responders to pegloticase compared to 30% of those under 60 (*P* = 0.015). Indeed, 61% of subjects who were 70 or older were responders. These data suggest that age may be a determinant of Ab formation and responsiveness. Whether this association relates to general age-related immunoinsufficiency, with a lesser capacity to generate T-cell help [22], or a greater likelihood to become tolerized to PEG with increasing age is unknown.

## Conclusions

Clinically significant anti-pegloticase Ab, present in 41% of patients, increased pegloticase clearance and did not neutralize uricase activity. Anti-pegloticase Ab were largely directed to the PEG portion of the molecule. Responders to pegloticase typically had low-titer Ab, whereas nonresponders usually developed high-titer Ab. Importantly, Ab titers at the time of study-visit infusions were not predictive of loss of sUA response, whereas loss of sUA response was predictive of IR risk. Patients with clinically significant anti-pegloticase Ab can be identified by loss of the sUA response, prompting treatment discontinuation.

## Abbreviations

Ab: antibodies; ELISA: enzyme-linked immunosorbent assay; Ig: immunoglobulin; IR: infusion reaction; IV: intravenous; mITT: modified intent-to-treat; PEG: polyethylene glycol; pUA: plasma urate; RCT: randomized placebo-controlled trial; sUA: serum uric acid; UA: uric acid.

## Competing interests

PL is a consultant for Savient Pharmaceuticals, Inc. LC is a consultant for Genentech, Pfizer, Bristol-Myers Squibb, Janssen, and Savient Pharmaceuticals, Inc. He is on the speaker bureau for Amgen and Genentech. AK receives grant/research support from Savient Pharmaceuticals Inc., Novartis, and Regeneron. JS receives research grants and is a consultant to Ardea (AstraZeneca), Regeneron, Metabolex, Pharmos, Savient Pharmaceuticals Inc., and Bristol-Myers Squibb. He receives research grants from Celgene and is a partner/owner in Academic Partners for Medical Education, LLC. DW is an employee of Savient Pharmaceuticals Inc. MW is a shareholder, previous employee, and current consultant for Savient Pharmaceuticals Inc. MB receives grant/research support from Savient Pharmaceuticals Inc. and Takeda. He is a consultant for Savient Pharmaceuticals Inc., Takeda, Ardea (AstraZeneca), BioCryst, Regeneron, Metabolex, and Sobi.

## Authors’ contributions

PL provided critical guidance on developing and evaluating the antibody assays and on interpreting the assay data and provided immunologic expertise in interpreting the results and drafting the manuscript. LC and AK participated in the analysis of the antibody data and provided expertise on immunologic implications of the findings. MB and JS participated in the clinical trials and the analysis of the antibody data and provided expertise on clinical implications of findings. DW supervised implementation of all assays and data collection and DW and MW participated in interpretation of the data. All authors made meaningful contributions to the drafting and content of the manuscript, provided critical review at multiple draft stages, and approved the submitted draft. All authors read and approved the final manuscript.

## Supplementary Material

Additional file 1 Supplementary methods. Click here for file

Additional file 2: Figure S1 Scattergrams showing individual Ab titer determinations at each study visit in all evaluable responders and nonresponders who received pegloticase biweekly and monthly. Click here for file

Additional file 3: Figure S2 Relationship between serum uric acid (sUA) levels and each category of anti-pegloticase antibody (Ab) titers. Click here for file

Additional file 4: Figure S3 Mean pre-dose anti- polyethylene glycol (PEG) antibody (Ab) titers over time for the pegloticase dosing groups by uric acid (UA) responder status. Click here for file

Additional file 5: Figure S4 Individual anti-uricase antibody (Ab) titer determinations for patients with positive anti-uricase Ab at any study visit during the randomized trials. Click here for file

Additional file 6: Figure S5 Individual patient profiles illustrate the relationships over time between serum uric acid (sUA) levels and anti-pegloticase antibodies for responders and nonresponders in each of the dosing groups. Patients were chosen as representative examples of dose and response type. Click here for file
