# Supplementary material for: Pegloticase immunogenicity: the relationship between efficacy and antibody development in patients treated for refractory chronic gout
Source: Arthritis Res Ther. 2014 Mar 4;16(2):R60. doi: 10.1186/ar4497 (PMC4060440; doi:10.1186/ar4497)
Supplement: Additional file 1 — Supplementary methods. [file ar4497-S1.doc]

**SUPPLEMENTARY METHODS**

**Summary of patient populations from the randomized trials**

Demographic and clinical characteristics were well balanced between trials and across treatment cohorts. Overall, the mITT population had a mean age of 55.4 years and consisted mostly of men (82%). Mean gout duration was 15 years; 73% of patients had tophi and 58% had chronic synovitis or arthropathy. Mean sUA was 9.8 mg/dL.

**ELISA assays**

For anti-pegloticase and anti-uricase assays, serum samples were diluted 1:30 with assay buffer and were added to microtiter plates coated with pegloticase or recombinant uricase as the capture antigen. For the detection of anti-PEG Ab, the serum samples were diluted 1:10 with assay buffer and assayed in microtiter plates coated with PEG. After the initial incubation and washing steps, horseradish peroxidase-conjugated goat anti-human IgG and anti-human IgM were added, and the presence of Ab was determined by spectrophotometry at 450 nm following addition of the peroxidase substrate. For determination of Ab titers, those serum samples found to contain detectable Ab were subjected to serial 3-fold dilutions in normal serum and were analyzed in duplicate. The final titer for each sample was defined as the highest dilution of serum that produced a mean absorbance (A450 nm) greater than the negative cut-off value.

For the uricase Ab assay, a rabbit anti-uricase Ab, affinity purified from antiserum obtained 7 weeks after uricase immunization, was used as a positive control. For the pegloticase Ab assay, serum containing high titers of IgG and IgM Ab to pegloticase obtained from a patient treated in an earlier clinical trial was used as a positive control during assay validation. The positive control for the anti-PEG assay was a monoclonal mouse anti-PEG antibody (Academia Sinica, Taiwan).

Validation of the ELISA assays included negative cut-off and cut-point factor determinations, as well as tests for intra- and inter-assay precision, sensitivity, specificity and recovery, stability (bench top, freeze-thaw cycles, long-term), drug interference, prozone effect and drug competition. The sensitivity of the anti-pegloticase Ab assay was 7.5 μg/mL for the enzyme portion and 23 ng/mL for the PEG moiety using purified rabbit anti-uricase and purified mouse anti-PEG positive controls. Sensitivity of the anti-uricase assay was 22.5 ng/mL. Pegloticase interference in undiluted human serum occurred at 300 µg/mL in the anti-pegloticase assay and 600 µg/mL in the anti-uricase assay. In the anti-PEG assay, interference occurred with 10 ng/mL pegloticase or 2.5 µg/mL PEG.

To determine the specificity of anti-pegloticase Ab, a variety of PEGylated proteins, including pegloticase (~40 PEGs/protein molecule; molecular weight 545 kDa), PEG-asparaginase (~40 PEGs/protein molecule; 340 kDa), PEG-catalase (~40 PEGs/protein molecule; 440 kDa), PEG-chymotrypsin (~9 PEGs/protein molecule; 70 kDa), PEG-subtilisin (~6 PEGs/protein molecule; 57 kDa) and PEG-superoxide dismutase (~10 PEGs/protein molecule; 82 kDa) were used in competition assays. All of the proteins except for pegloticase were modified with 5 kDa PEG and were from Sigma-Aldrich (St Louis, Missouri). In this assay, soluble pegloticase (or other soluble PEGylated protein) competed with pegloticase coated on the ELISA plate for binding to anti-pegloticase Ab in the serum. Serum samples from a phase 2 clinical trial patient that were positive for anti-pegloticase Ab were used for this assay. Samples were obtained at times when this subject had both low titer and high titer anti-pegloticase Ab. Samples were diluted 1:30 and assayed for Ab in pegloticase-coated ELISA wells in the presence or absence of 200 µg/mL of the various soluble PEGylated proteins. A mixture of lysozyme (50 µg/mL) and propylene oxide (150 µg/mL) was used as a negative control. To confirm the specificity of the anti-uricase Ab assay, study samples were diluted 1:30 before addition of uricase 2 µg/mL (or 2 µg/mL lysozyme for the negative control).

**Determination of pegloticase concentrations**

An enzymatic/fluorescence assay was used to quantitate pegloticase concentrations in serum. In the assay, pegloticase catalyzes the conversion of UA to allantoin, thus releasing hydrogen peroxide (H_2_O_2_) and carbon dioxide. In the presence of horseradish peroxidase, H_2_O_2_ reacts with a 1:1 stoichiometry with Amplex Ultra Red (Molecular Probes, Inc., Eugene, Oregon) to generate the red fluorescence oxidation product, resorufin. The concentration of resorufin, which is determined by flourimetry, is proportional to the amount of active pegloticase present in the serum samples. The lower limit of detection was determined to be 0.6 µg/mL.
