# Supplementary figures and images for: Pegloticase immunogenicity: the relationship between efficacy and antibody development in patients treated for refractory chronic gout
Source: Arthritis Res Ther. 2014 Mar 4;16(2):R60. doi: 10.1186/ar4497 (PMC4060440; doi:10.1186/ar4497)

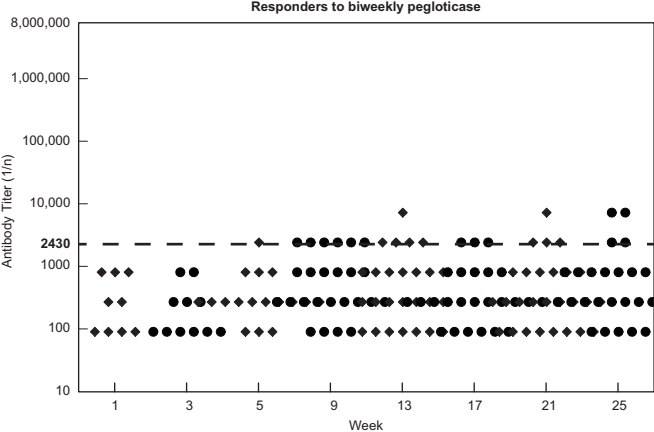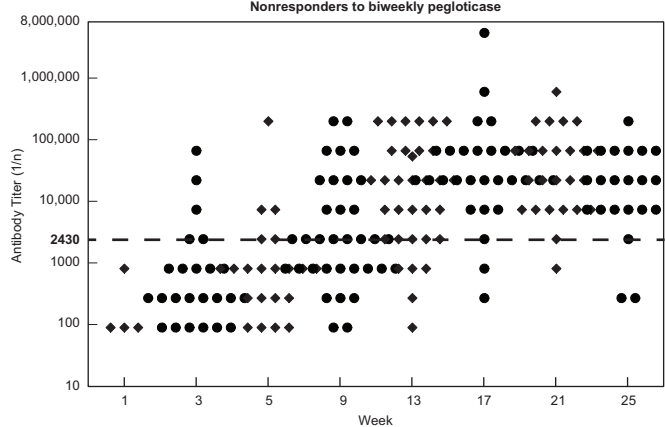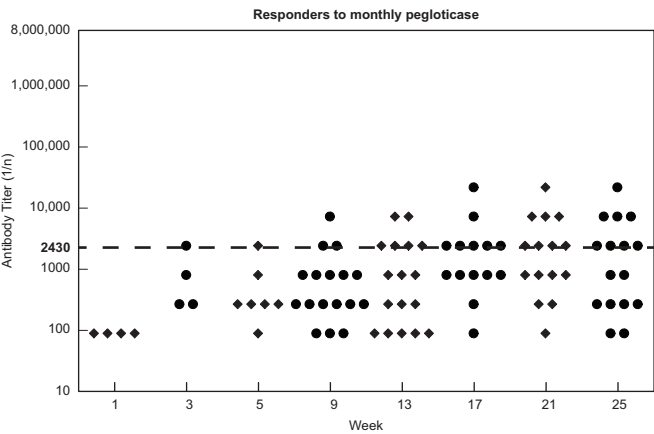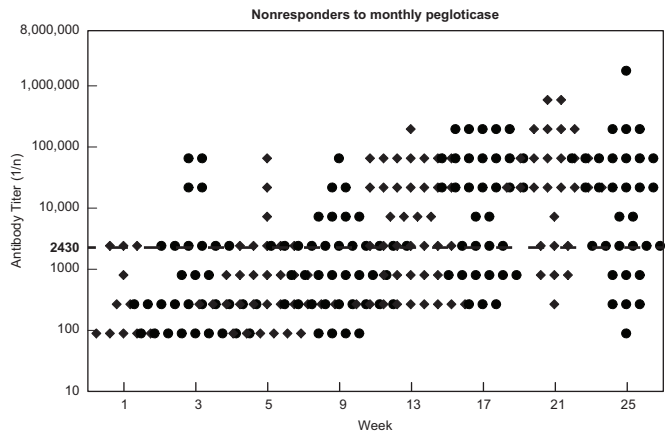

Supplement: Additional file 2: Figure S1 — Scattergrams showing individual Ab titer determinations at each study visit in all evaluable responders and nonresponders who received pegloticase biweekly and monthly. [file ar4497-S2.pdf]

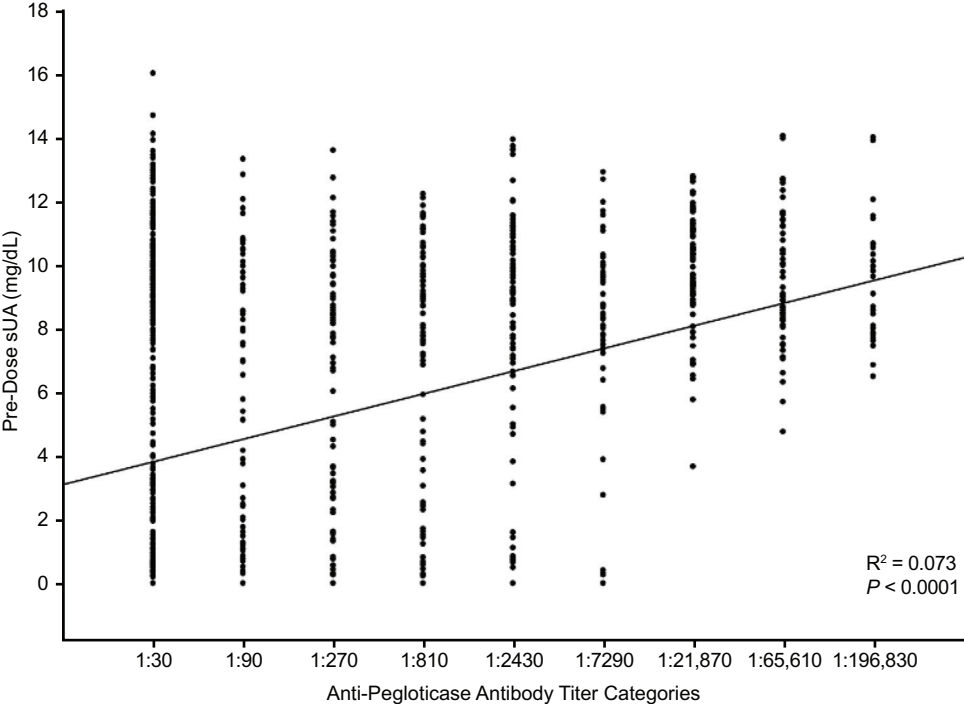

Supplement: Additional file 3: Figure S2 — Relationship between serum uric acid (sUA) levels and each category of anti-pegloticase antibody (Ab) titers. [file ar4497-S3.pdf]

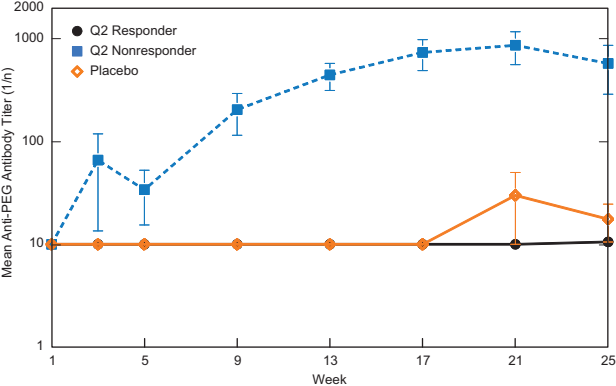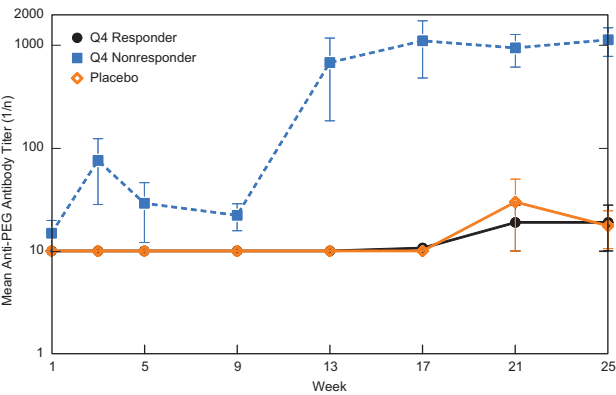

Supplement: Additional file 4: Figure S3 — Mean pre-dose anti- polyethylene glycol (PEG) antibody (Ab) titers over time for the pegloticase dosing groups by uric acid (UA) responder status. [file ar4497-S4.pdf]

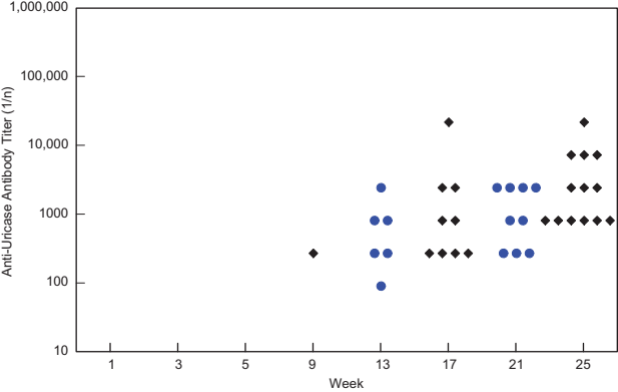

Supplement: Additional file 5: Figure S4 — Individual anti-uricase antibody (Ab) titer determinations for patients with positive anti-uricase Ab at any study visit during the randomized trials. [file ar4497-S5.pdf]

**Subject A (q2, responder)**

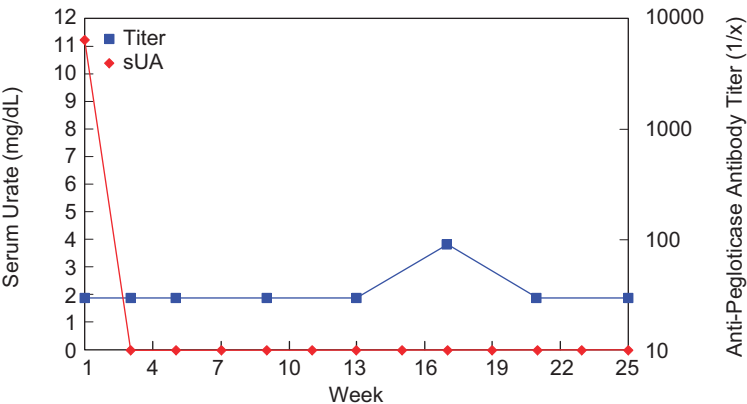

**Subject C (q2, nonresponder)**

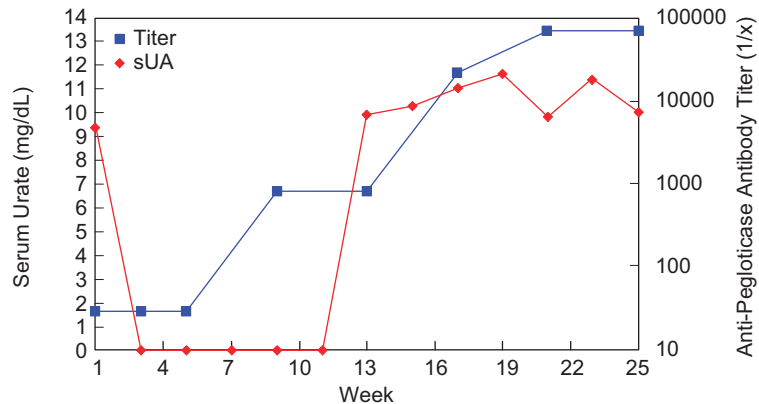

**Subject D (q4, responder)**

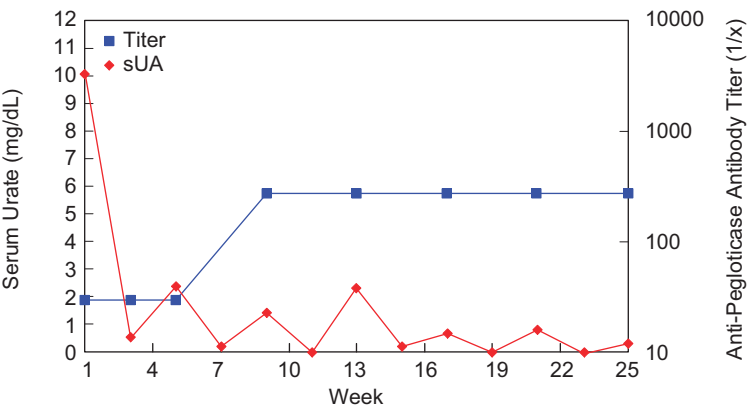

**Subject E (q4, nonresponder)**

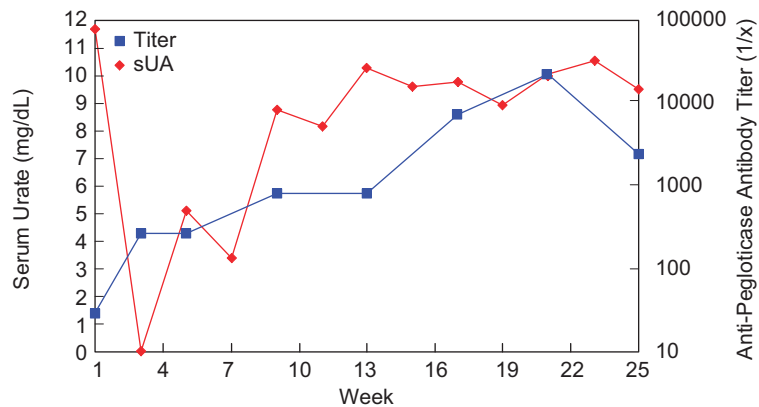

Supplement: Additional file 6: Figure S5 — Individual patient profiles illustrate the relationships over time between serum uric acid (sUA) levels and anti-pegloticase antibodies for responders and nonresponders in each of the dosing groups. Patients were chosen as representative examples of dose and response type. [file ar4497-S6.pdf]
